# Calcium Chloride and Calcium Gluconate in Neonatal Parenteral Nutrition Solutions without Cysteine: Compatibility Studies Using Laser Light Obscuration Methodology

**DOI:** 10.3390/nu10020208

**Published:** 2018-02-14

**Authors:** Robert K. Huston, J. Mark Christensen, Sultan M. Alshahrani, Sumeia M. Mohamed, Carl F. Heisel

**Affiliations:** 1 Northwest Newborn Specialists, PC and Pediatrix Medical Group, Portland, OR 97227, USA; 2 Department of Pharmaceutical Sciences, College of Pharmacy, Oregon State University, Corvallis, OR 97331, USA; jmark.christensen@oregonstate.edu (J.M.C.); alshahrs@onid.oregonstate.edu (S.M.A.); mohameds@onid.oregonstate.edu (S.M.M.); 3 Neonatal Pharmacy, Randall Children’s Hospital at Legacy Emanuel, Portland, OR 97227, USA; CHeisel@lhs.org

**Keywords:** calcium chloride, calcium gluconate, compatibility, neonates, parenteral nutrition

## Abstract

There are no compatibility studies for neonatal parenteral nutrition solutions without cysteine containing calcium chloride or calcium gluconate using light obscuration as recommended by the United States Pharmacopeia (USP). The purpose of this study was to do compatibility testing for solutions containing calcium chloride and calcium gluconate without cysteine. Solutions of TrophAmine and Premasol (2.5% amino acids), containing calcium chloride or calcium gluconate were compounded without cysteine. Solutions were analyzed for particle counts using light obscuration. Maximum concentrations tested were 15 mmol/L of calcium and 12.5 mmol/L of phosphate. If the average particle count of three replicates exceeded USP guidelines, the solution was determined to be incompatible. This study found that 12.5 and 10 mmol/L of calcium and phosphate, respectively, are compatible in neonatal parenteral nutrition solutions compounded with 2.5% amino acids of either TrophAmine or Premasol. There did not appear to be significant differences in compatibility for solutions containing TrophAmine or Premasol when solutions were compounded with either CaCl_2_ or CaGlu-Pl. This study presents data in order to evaluate options for adding calcium and phosphate to neonatal parenteral nutrition solutions during shortages of calcium and cysteine.

## 1. Introduction

Neonatal parenteral nutrition solutions compounded with calcium chloride (CaCl_2_) and calcium gluconate in plastic vials (CaGlu-Pl) have low concentrations of aluminum (Al) compared to solutions compounded with calcium gluconate in glass vials (CaGlu-Gl) [1,2,3]. The adverse effects associated with exposure of preterm infants to high Al concentrations in parenteral nutrition (PN) include neurodevelopmental impairment and decreased bone mineralization [1,4]. While CaGlu-Pl has been available in Europe for some time [2], this low aluminum containing additive has only recently become available in North America. If there is a shortage of CaGlu-Pl, CaCl_2_ is the only calcium additive that is an option in order to limit aluminum intake to recommended levels [3]. There has been reluctance to compound PN solutions with CaCl_2_ due to the decreased compatibility of CaCl_2_ with mineral phosphates compared to the compatibility of mineral phosphates with calcium gluconate [5,6]. Recent studies using laser light dynamic light scattering as well as micro-flow imaging (MFI) methodology have provided data with regard to compatible concentrations of CaCl_2_ with mineral phosphates [7,8]. Laser light dynamic light scattering is able to provide data on the particle size distribution but does not measure actual particle counts while MFI has been the preferred method to evaluate proteinaceous particles; therefore, the preferred method recommended by the United States Pharmacopeia (USP) for evaluating calcium phosphate particle formation is laser light obscuration methodology [9]. A literature search has not found previously published compatibility studies using laser light methodology for neonatal PN solutions containing calcium gluconate and mineral phosphates without added cysteine. Recurrent shortages of calcium and cysteine additives make it necessary to evaluate different options when various additives of calcium and cysteine are not available.

The objective of this study was to evaluate the compatibility of CaCl_2_ with phosphate in neonatal PN solutions without added cysteine using laser light obscuration (LO) methodology for concentrations of calcium and phosphate that appeared to be compatible in previous studies using laser light dynamic light scattering and MFI methodologies. Studies of solutions containing CaGlu-Pl were also conducted. 

## 2. Methods

The study was approved by the Institutional Review Board for the hospital system. All study solutions were compounded by a neonatal pharmacist in the children’s hospital pharmacy in clear plastic Exacta Mix 250 mL EVA containers (Baxter Healthcare, Deerfield, IL, USA) using a Baxa Exactamix 2400 Compounder (Baxa Corporation, Englewood, CO, USA). Additives that were included in all solutions are shown in Table 1. The final volume of each solution was 200 mL.

Studies were conducted for solutions containing 2.5% amino acids (AA). This concentration of AA was chosen because a concentration of ≥2.5% AA is most often used clinically in the newborn intensive care unit (NICU). Studies were conducted using TrophAmine^®^ (B. Braun Medical Inc., Bethlehem, PA, USA) and repeated using Premasol^®^ (Baxter Healthcare, Deerfield, IL, USA) as the AA additive. Calcium chloride (IMS Ltd., So. El Monte, CA, USA) or CaGlu-Pl (Fresenius Kabi USA, Lake Zurich, IL, USA) was added in concentrations of 7.5, 10, 12.5, and 15 mmol/L. Each concentration of calcium was compounded with phosphate in concentrations of 7.5, 10, and 12.5 mmol/L. We intended to use sodium phosphate (Hospira, Lake Forest, IL, USA) but due to the shortage of this additive, some solutions were compounded with potassium phosphate (Fresenius Kabi USA, Lake Zurich, IL, USA). Control solutions containing no calcium and no phosphate, and containing 12.5 mmol/L of calcium and no phosphate were compounded for studies of both CaCl_2_ and CaGlu-Pl. All solutions were compounded in triplicate.

After compounding, solutions were incubated at 37 °C for 24 h in a warming oven. Bags were visualized in a dark room while trans-illuminating the solution with a bright beam of light before and after incubation to determine evidence of precipitation. Solution pH was measured before and after incubation using a pH meter (Mettler Toledo FIVE^TM^, Schwerzenbach, Switzerland). Particle counts determined by laser LO with an Accusizer 780 SIS instrument (Particle Sizing Systems, Port Richey, FL, USA) and microscopic particle counts (Celestron Microscope pro Model 44308, Torrance, CA, USA) were performed using procedures recommended by the USP Chapter 788. For LO, suggested limits for particles ≥ 10 microns are 25 particles per mL, and for particles ≥ 25 microns are 3 particles per mL. For microscopic analysis, suggested limits for particles ≥ 10 microns are 12 particles per mL, and for particles ≥ 25 microns are 2 particles per mL. Average particle counts of the three solutions compounded with the same calcium and phosphate concentrations were calculated. Solutions which had an average particle count exceeding USP Chapter 788 guidelines were considered incompatible.

## 3. Results

### 3.1. Solution pH

The pH of study solutions is shown in Table 2 and Table 3 for solutions compounded with TrophAmine and Premasol, respectively. There were no significant differences in pH related to calcium concentration, therefore, the pH of all solutions containing any calcium were averaged for a given phosphate concentration. Solutions containing Premasol had lower pH compared to TrophAmine. The pH of all solutions decreased after incubation at 37 °C for 24 h. Adding calcium either as CaCl_2_ or as CaGlu had a minimal effect on pH.

### 3.2. Visual Studies

No solution had visual evidence of precipitation in the dark room. Microscopic particle counts for particles ≥ 10 microns and for particles ≥ 25 microns were <1 particle/mL for all solutions.

### 3.3. Light Obscuration

Particle counts for particles ≥ 10 microns, as determined by LO, are displayed in Table 4 and Table 5 for TrophAmine and Premasol, respectively. Since there were three replicates for each solution containing the same concentrations of additives, the minimum, median, and maximum particle counts are listed. Incompatible solutions are noted in the tables. There were minimal differences between solutions made with Premasol versus TrophAmine.

For particle counts ≥ 25 microns, only three solutions, all compounded with TrophAmine, were incompatible based on USP guidelines. All of these solutions had average particle counts for particles ≥ 10 microns that were incompatible using USP guidelines. There were five control solutions containing no calcium or phosphate for TrophAmine. The minimum, median, and maximum particle counts for particles ≥10 microns were 1, 7, and 14 particles per mL, respectively, and for particles ≥ 25 microns were 0, 0, and 2 particles per mL. For Premasol there were six control solutions that contained no calcium or phosphate. The minimum, median, and maximum particle counts for particles ≥ 10 microns were 0, 3, and 10 particles per mL, respectively, and for particles ≥ 25 microns were 0, 0 and 0 particles per mL.

## 4. Discussion

Previous studies of the compatibility of CaCl_2_ with mineral phosphate in neonatal PN solutions containing at least 2.5% AA and no cysteine found that the maximum amount of phosphate that was compatible with 10 mmol/L of calcium was 7.5 mmol/L [7,8]. The current study using LO and USP criteria for compatibility found that 12.5 mmol/L of either calcium chloride or calcium gluconate and 10 mmol/L of phosphate were compatible in parenteral nutrition solutions containing both TrophAmine and Premasol. The greater compatibility of CaCl_2_ with mineral phosphate in this study compared to previous studies may be related to subtle differences due to compounding at different times and unavoidable variability in the compounding process itself. There also may be some variability in the concentrations of individual AAs due to using different lots of AA additives in the different studies. The pH of solutions used in the studies was quite similar, however. The other variable was the methods used to determine compatibility. There is data in the literature that suggests that micro-flow imaging may be more accurate than LO in determining the size of non-spherical particles such as may be formed in calcium phosphate precipitates [10,11,12]. A study using both methods to determine particle counts of PN solutions has not been performed. However, the microscopic procedure recommended by USP was used in this study and in the previous study that employed MFI to determine compatibility.

Minimal recommended parenteral calcium and phosphate intakes have been 1.5 mmol/kg/day (60 mg/kg/day and 47 mg/kg/day, respectively) [13]. The PN fluid intake required to deliver this minimum intake of both calcium and phosphate for solutions containing 12.5 mmol/L of calcium and 10 mmol/L of phosphate, concentrations found to be compatible in this study, would be 150 mL/kg/day. Higher concentrations of calcium and phosphate have been found to be compatible when adding cysteine to solutions containing mineral phosphates [14] as well as when replacing mineral phosphate with organic phosphate [15]. Organic phosphates are not approved for routine use in North or Central America, and there are no studies using LO that have been published on the compatibility of CaCl_2_ and mineral phosphates for solutions containing cysteine. There is also little data in the literature using LO to determine the compatibility of CaGlu and mineral phosphates in neonatal PN solutions containing cysteine, and the data that is published appear to show particle counts that exceed USP guidelines [16,17]. This study evaluated solutions without added cysteine in order to provide data to evaluate options when cysteine is unavailable in the United States, where cysteine is added separately to PN solutions. Although the neonatal AA preparation used in Europe (Primene, Baxter, Lintec, France, Belgium) contains cysteine, we are not aware of any studies using LO to measure particle counts to determine compatibility of calcium salts and phosphates in solutions containing this AA preparation.

Similar to a previous study [7], there were minimal differences in compatibility for solutions compounded with TrophAmine versus Premasol even though the pH of solutions compounded with Premasol was lower than for solutions compounded with TrophAmine. The AA concentrations of individual AAs are almost identical in the two products which may explain the similar findings for compatibility.

This is the first study to evaluate the compatibility of calcium gluconate and mineral phosphate in neonatal PN solutions without cysteine containing AA concentrations often used in clinical care with LO methods as recommended by the USP. A previous study, using LO to determine compatibility of organic and inorganic calcium and phosphates, evaluated solutions containing low concentrations of dextrose and AA that are rarely used in clinical practice [15]. In the current study, there were minimal differences in compatibility between solutions containing CaCl_2_ and calcium gluconate. Higher amounts of CaGlu-Pl than CaCl_2_ were shown to be compatible with some phosphate concentrations as expected.

There are limitations to this study. Due to the shortage of sodium phosphate, some solutions were compounded with potassium phosphate. At the pH of solutions tested in this study, dissociation of K^+^ and Na^+^ from monobasic and dibasic phosphate is almost complete and the primary determinants of compatibility are the concentrations of the phosphate and calcium ions [18]. One concentration equal to 2.5% AA was used in this study. Solutions used clinically in our NICU often have AA concentrations of 2.5–3% even though the dose of AA ranges from 3–4 g/kg/day. Previous studies have not found a significant difference in compatible amounts of calcium and phosphate between solutions containing 2.5 and 3% AA compounded with [8,15] or without [8,9] added cysteine using other methods than LO to measure particle counts; this is why only solutions containing 2.5% AA were included in this study.

## 5. Conclusions

In conclusion, this study using laser LO methodology as recommended by USP Chapter 788 found that 12.5 and 10 mmol/L of calcium and phosphate, respectively, are compatible in neonatal PN solutions compounded with 2.5% AA of either TrophAmine or Premasol. There did not appear to be significant differences in compatibility for solutions containing TrophAmine or Premasol when solutions were compounded with either CaCl_2_ or CaGlu-Pl. This is the first study to provide data on the compatibility of CaCl_2_ and CaGlu with mineral phosphates in solutions compounded without cysteine for neonatal PN solutions used clinically based upon USP criteria for compatibility. The study adds data to the literature which may be used to evaluate different options for administering calcium and phosphate in neonatal PN. This may be valuable information during times of shortage of PN additives of calcium and cysteine. Further studies of neonatal PN solutions containing cysteine using LO to determine compatibility of calcium salts with phosphate are needed.

## Figures and Tables

**Table 1:** Additives included in all study solutions.

Additive	Manufacturer	Dose/dL
Dextrose 70%	Baxter Healthcare, Deerfield, IL, USA	10 g
Sodium Acetate (2 mEq/mL)	Hospira Inc., Lake Forest, IL, USA	2 mEq
Potassium Chloride (2 mEq/mL)	Hospira Inc., Lake Forest, IL, USA	1 mEq
Magnesium Sulfate 50%	APP Pharmaceuticals, Schaumburg, IL, USA	0.5 mEq
Heparin (100 units/mL)	BD Pharmaceuticals, Schaumburg, IL, USA	50 units
Zinc Chloride (1 mg/mL)	Hospira Inc., Lake Forest, IL, USA	400 mcg
Copper (40 mcg/mL)	Hospira Inc., Lake Forest, IL, USA	20 mcg
l-Carnitine (20 mg/mL)	Sigma-Tau, Gaithersburg, MD, USA	5 mg
Water	Baxter Healthcare, Deerfield, IL, USA	qs

**Table 2:** Mean and Standard Deviation for pH of solutions compounded with TrophAmine (*N* = number of bags).

*N*	Calcium	Phosphate	0 h	24 h
5	none	0 mmol/L	5.71 + 0.09	5.59 + 0.03
3	Chloride	0 mmol/L	5.64 + 0.04	5.54 + 0.03
12	Chloride	7.5 mmol/L	5.75 + 0.03	5.69 + 0.03
12	Chloride	10 mmol/L	5.78 + 0.03	5.69 + 0.02
12	Chloride	12.5 mmol/L	5.79 + 0.02	5.73 + 0.02
3	Gluconate	0 mmol/L	5.68 + 0.04	5.60 + 0.02
12	Gluconate	7.5 mmol/L	5.75 + 0.03	5.70 + 0.02
12	Gluconate	10 mmol/L	5.80 + 0.02	5.73 + 0.02
12	Gluconate	12.5 mmol/L	5.83 + 0.03	5.76 + 0.04

**Table 3:** Mean and Standard Deviation for pH of solutions compounded with Premasol (*N* = number of bags).

*N*	Calcium	Phosphate	0 h	24 h
6	none	0 mmol/L	5.57 + 0.01	5.51 + 0.03
3	Chloride	0 mmol/L	5.56 + 0.02	5.47 + 0.05
12	Chloride	7.5 mmol/L	5.63 + 0.01	5.56 + 0.03
12	Chloride	10 mmol/L	5.66 + 0.01	5.58 + 0.03
12	Chloride	12.5 mmol/L	5.55 + 0.01	5.48 + 0.01
3	Gluconate	0 mmol/L	5.58 + 0.01	5.53 + 0.01
12	Gluconate	7.5 mmol/L	5.64 + 0.01	5.58 + 0.02
12	Gluconate	10 mmol/L	5.67 + 0.01	5.60 + 0.03
12	Gluconate	12.5 mmol/L	5.69 + 0.01	5.63 + 0.02

**Table 4:** Particle counts ≥ 10 microns per mL ^a^ for solutions compounded with TrophAmine (*N* = number of bags).

*N*	Calcium ^b^	Phosphate	Chloride	Gluconate
3	12.5 mmol/L	0 mmol/L	(1, 2, 7)	(1, 13, 27)
3	7.5 mmol/L	7.5 mmol/L	(3, 13, 21)	(5, 7, 14)
3	10 mmol/L	7.5 mmol/L	(1, 4, 9)	(1, 12, 30)
3	12.5 mmol/L	7.5 mmol/L	(1, 3, 22)	(1, 2, 45)
3	15 mmol/L	7.5 mmol/L	(29, 36, 51) ^c,d^	(1, 1, 3) ^d^
3	7.5 mmol/L	10 mmol/L	(3, 4, 7)	(2, 2, 54)
3	10 mmol/L	10 mmol/L	(2, 5, 8)	(3, 4, 21)
3	12.5 mmol/L	10 mmol/L	(2, 2, 4)	(1, 13, 21)
3	15 mmol/L	10 mmol/L	(26, 31, 42) ^c,d^	(26, 29, 33) ^c,d^
3	7.5 mmol/L	12.5 mmol/L	(6, 12, 18) ^d^	(0, 23, 35)
3	10 mmol/L	12.5 mmol/L	(2, 3, 9) ^d^	(0, 11, 14)
3	12.5 mmol/L	12.5 mmol/L	(25, 36, 48) ^c,d^	(8, 14, 19)
3	15 mmol/L	12.5 mmol/L	(50, 62, 81) ^c,d^	(29, 32, 40) ^c,d^

^a^ Minimum, median, maximum; ^b^ one mmol of calcium = 40 mg or 2 mEq; ^c^ highlighted solutions have average particle counts that exceed United States Pharmacopeia Standards; ^d^ compounded with potassium phosphate versus sodium phosphate.

**Table 5:** Particle counts ≥ 10 microns per mL ^a^ for solutions compounded with Premasol (*N* = number of bags).

*N*	Calcium ^b^	Phosphate	Chloride	Gluconate
3	12.5 mmol/L	0 mmol/L	(1, 2, 2)	(1, 4, 12)
3	7.5 mmol/L	7.5 mmol/L	(1, 2, 24)	(0, 1, 4)
3	10 mmol/L	7.5 mmol/L	(1, 1, 23)	(1, 2, 2)
3	12.5 mmol/L	7.5 mmol/L	(1, 2, 3)	(1, 2, 2)
3	15 mmol/L	7.5 mmol/L	(29, 34, 40) ^c,d^	(16, 18, 22) ^d^
3	7.5 mmol/L	10 mmol/L	(0, 2, 3)	(1, 2, 7)
3	10 mmol/L	10 mmol/L	(1, 5, 7)	(1, 2, 5)
3	12.5 mmol/L	10 mmol/L	(1, 1, 6)	(1, 1, 1)
3	15 mmol/L	10 mmol/L	(45, 50, 69) ^c,d^	(23, 25, 29) ^c,d^
3	7.5 mmol/L	12.5 mmol/L	(9, 12, 18) ^d^	(0, 1, 19)
3	10 mmol/L	12.5 mmol/L	(17, 19, 21) ^d^	(1, 3, 15)
3	12.5 mmol/L	12.5 mmol/L	(24, 27, 30) ^c,d^	(2, 4, 185) ^c^
3	15 mmol/L	12.5 mmol/L	(37, 66, 85) ^c,d^	(26, 30, 41) ^c,d^

^a^ Minimum, median, maximum; ^b^ one mmol of calcium = 40 mg or 2 mEq; ^c^ highlighted solutions have average particle counts that exceed United States Pharmacopeia Standards; ^d^ compounded with potassium phosphate versus sodium phosphate.
